# The Role of Emotional Expression and Eccentricity on Gaze Perception

**DOI:** 10.3389/fpsyg.2019.01129

**Published:** 2019-05-21

**Authors:** Deema Awad, Nathan J. Emery, Isabelle Mareschal

**Affiliations:** Department of Biological and Experimental Psychology, School of Biological and Chemical Sciences, Queen Mary University of London, London, United Kingdom

**Keywords:** gaze, faces, emotions, peripheral vision, contextual effects

## Abstract

The perception of another’s gaze direction and facial expression complements verbal communication and modulates how we interact with other people. However, our perception of these two cues is not always accurate, even when we are looking directly at the person. In addition, in many cases social communication occurs within groups of people where we can’t always look directly at every person in the group. Here, we sought to examine how the presence of other people influences our perception of a target face. We asked participants to judge the direction of gaze of the target face as either looking to their left, to their right or directly at them, when the face was viewed on its own or viewed within a group of other identity faces. The target face either had an angry or a neutral expression and was viewed directly (foveal experiment), or within peripheral vision (peripheral experiment). When the target was viewed within a group, the flanking faces also had either neutral or angry expressions and their gaze was in one of five different directions (from averted leftwards to averted rightwards in steps of 10°). When the target face was viewed foveally there was no effect of target emotion on participants’ judgments of its gaze direction. There was also no effect of the presence of flankers (regardless of expression) on the perception of the target gaze. When the target face was viewed peripherally, participants judged its direction of gaze to be direct over a wider range of gaze deviations than when viewed foveally, and more so for angry faces than neutral faces. We also find that flankers (regardless of emotional expression) did not influence performance. This suggests that observers judge that angry faces were looking at them over a broad range of gaze deviations in the periphery only, possibly resulting from increased uncertainty about the stimulus.

## Introduction

We are sensitive to a variety of different facial cues during successful communication (Simpson and Crandall, 1972; Ekman, 1993; Engell and Haxby, 2007). Of the non-verbal cues that people exchange and decipher during these interactions, both eye gaze and facial expressions rapidly capture our attention, providing us with information about another’s emotional state, focus of attention, intentions, and future behavior (Vuilleumier, 2005; Palermo and Rhodes, 2007).

From an early age (see McClure, 2000 for meta-analysis on emotional recognition from infancy to adolescence), and regardless of gender (Thayer and Johnsen, 2000), or cultural background (Ekman and Wallace, 1971), humans are experts in analyzing others’ facial emotional expressions, which serve as a reliable indicator of their internal emotional and mental states (Adolphs, 2003). From an evolutionary perspective, the rapid and accurate detection of facial expressions is seen as critical for survival (Darwin, 1872). This is supported by EEG studies demonstrating faster cortical activity during the perception of emotional faces compared to neutral faces, highlighting the role of the right dorsolateral prefrontal cortex in emotional processing (Prete et al., 2015; Prete et al., 2018). Support for the rapid detection and accurate perception of facial expression also comes from behavioral studies showing that negative facial emotions, (notably those eliciting feelings of threat and danger) draw attention quickly and involuntarily (Öhman et al., 2001; Armony and Dolan, 2002; Holmes et al., 2003), and that angry faces “pop out” in a crowd (Hansen and Hansen, 1988; Feldmann-Wüstefeld et al., 2011), even when the emotional content is not relevant to the task (Frühholz et al., 2011), or is viewed under conditions of restricted awareness (Mogg and Bradley, 1999). Rigoulot et al. (2012) proposed that the influence of facial expressions on behavior can result from two opposing sources. First, emotional expressions could improve performance by automatically capturing attention and directing it to the relevant part of the task, leading to improved performance, such as has been demonstrated in dot-probe paradigms (Armony and Dolan, 2002; Brosch et al., 2007). Second, emotional expressions could disrupt the processing of task-relevant information if participants struggle to disengage from the emotional stimuli, leading to impaired performance as occurs for example, in emotional Stroop tasks (Williams et al., 1996; Fox et al., 2001; Frühholz et al., 2011; Rigoulot et al., 2012).

The accurate perception of another’s gaze direction is also essential to social interactions, providing important information about their focus of attention, future intentions, as well as their emotional states (Emery, 2000; Baron-Cohen et al., 2001; Calder et al., 2008; Mareschal et al., 2013a,b, 2014). As with the detection of facial emotions, the relevance of gaze has been demonstrated from a very early age. For example, infants and monkeys as young as 3 or 4 months old follow the eye gaze direction of others and regulate their own attention appropriately (Moore and Corkum, 1998; Ferrari et al., 2000). Although the direction of the eyes is important in judging gaze, converging evidence reveals that people use a combination of cues to judge where another person is looking, using information from the pupil and sclera (Kobayashi and Kohshima, 2001), head orientation (Langton, 2000; Otsuka et al., 2014), torso direction (Seyama and Nagayama, 2005), and their prior expectations (Mareschal et al., 2013a). In order to quantify how sensitive we are to another person’s gaze, Gamer and Hecht (2007) measured the range of gaze deviations that an individual perceives as being directed toward them [often referred to as the cone of direct gaze (CoDG)]. This CoDG measure is approximately 8–10° in adults (e.g., Gamer and Hecht, 2007; Mareschal et al., 2013b; Florey et al., 2015), and is wider in children (Vida and Maurer, 2012; Mareschal et al., 2016). Recently Mareschal et al. (2013b) developed a CoDG model that quantifies the changes in gaze perception performance. The model accounts for performance with three parameters: (1) the peak of the CoDG or the bias of perceived direct gaze (the gaze deviation the participants judge most as being direct; in case of no bias, this value would be 0). (2) the width of the cone of direct gaze which is calculated as the distance between the category boundaries for direct and averted gaze deviations; which is the range of gaze deviations participants judged as being direct (3) the estimate of the internal noise (or uncertainty) associated with the perception of gaze. This parameter reflects the amount of uncertainty associated with the observers’ internal representation of the gaze direction (see section Materials and Methods for more details).

In addition to cues from the eyes and head, our perception of where another person is looking can be influenced by secondary cues, such as facial expression (Mathews et al., 2003; Putman et al., 2006; Lobmaier et al., 2008; Ewbank et al., 2009). For example, Ewbank et al. (2009) asked their participants to judge the direction of gaze in angry, fearful and neutral faces. They found that the CoDG was wider when they used angry faces compared to fearful and neutral faces. Putman et al. (2006) investigated whether reflexive cueing of attention that occurs after perception of a gaze cue is greater for fearful than for happy faces in normal and anxious participants. They used a dynamic stimulus presentation displaying faces that simultaneously morphed from a neutral into a happy or fearful expression and whose eye gaze shifted from direct to averted while participants performed a cueing task. They found that fearful faces induced stronger gaze cueing than happy faces, and that the strength of this cueing effect was correlated with participants’ anxiety levels. Additionally, Mathews et al. (2003) investigated whether a fearful expression enhances the effect of another’s gaze in directing the attention of the observer. Anxious and non-anxious participants viewed faces with either direct or averted gaze, and the participants’ task was to locate target letters in the display. They found a difference between the two groups. Notably, attention was guided by the direction of gaze in fearful faces more so than in neutral faces, but only in the anxiety-prone individuals. Interestingly, these interactive effects of gaze and expression also seem present from an early age. Striano et al. (2006) measured larger amplitude event-related potentials from the scalps of 4 months old infants when they were presented with angry faces whose gaze was direct than when presented with angry faces whose gaze was averted.

Since both gaze and facial expressions can elicit rapid and automatic spatial orienting, this has led many to examine whether there are joint brain regions facilitating the perception of both types of facial cues. In a fMRI study, Engell and Haxby (2007) presented participants with neutral faces with either direct-gaze or averted-gaze, or emotionally expressive faces with direct-gaze. The authors found that the inferior occipital gyrus, fusiform gyrus, superior temporal sulcus (STS) and inferior frontal gyrus were strongly activated in the emotionally expressive faces conditions, and the right STS was more strongly activated in the averted-gaze than in the direct-gaze conditions. Further comparisons of the data in the right STS demonstrated that emotional expression and averted gaze activated distinct, though overlapping cortical regions in the STS. Thus, the authors argued that gaze-direction and expression are associated with dissociable but overlapping neural systems, and that the overlapping regions might be responsible for the integration of emotional expression and gaze-direction information.

Behavioral studies have also demonstrated the influence that eye-gaze has on the recognition of facial emotional expression (Adams and Kleck, 2003, 2005). For example, Adams and Kleck (2003) found that participants recognized angry and happy faces more quickly when the face had a forward facing (direct) gaze, whereas fearful and sad faces were categorized more quickly when their gaze was averted. In another study, Adams and Kleck (2005) asked participants to classify the emotion (anger, joy, fear, sadness) of faces with different eye gaze (averted or direct), and found that direct gaze enhanced the perception of approach-oriented emotions (anger and joy) and averted gaze enhanced the perception of avoidance-oriented emotions (fear and sadness). They argue that combining information about gaze and expression is critical for survival since this provides us with information that someone intends to approach (or avoid) us so that we react appropriately to them.

Most of the above studies examined the perception of faces (their direction of gaze or facial emotion) when they were viewed on their own. However, it is well established that a stimulus (for example, a face) can appear differently when surrounded by other objects than when viewed on its own (Palmer, 1975; Levitt and Lund, 1997; Todorović, 2010). For example, Walker and Vul (2014) showed participants photographs of women viewed either in a group or in isolation and found that participants judged the (same) woman to be significantly more attractive when she was part of a group than when viewed alone. They concluded that this was the result of the visual system averaging visual information (about the faces), resulting in a prototypical (more attractive) face. A different study by Haberman and Whitney (2009) investigated contextual effects on face perception. They presented participants with sets of faces varying in emotionality (e.g., happy to sad) followed by a test face. The participants’ task was to either indicate whether the test face had been a member of the previously viewed set of faces, or whether the test face looked happier or sadder than the average of the set of faces. They found that participants were unable to determine whether the test face had been in the original set of faces, although they were able to judge whether it looked happier or sadder than the average of the set. Thus, the authors argued that although participants retained little information about the individual members of the set, they had a remarkable representation of the mean emotion of the set of faces, due to “ensemble averaging.” For stimuli viewed in the periphery, averaging can be compulsory due to crowding whereby individual objects that are identifiable on their own become difficult to discriminate when presented with other objects. The strength of crowding depends on the similarity of the objects. For example, Parkes et al. (2001) presented participants with an oriented Gabor patch on its own or surrounded by another 8 Gabor patches of different orientations, and found that participants were unable to report the orientation of the central Gabor, although they were able to report the average orientation of the group. Thus, the authors argued that there is compulsory averaging of visual information in the periphery, whereby groups of (spatially proximal) objects are processed as an average rather than individually.

A question that arises from this is how peripheral vision affects our ability to process other types of information about faces? Bayle et al. (2009) investigated the processing of facial expressions for stimuli presented in the parafovea using magnetoencephalography. They recorded brain activity in response to centrally and parafoveally presented fearful faces and found that when the face was in the periphery, there was increased neural activation in the amygdala and fusiform gyrus when the face was fearful compared to when it was neutral, and that these faces were processed faster. Another study by Rigoulot et al. (2011) also investigated the impact of fearful faces when presented in the near and far periphery. Reaction times and event related potentials (ERPs) were recorded while participants were asked to categorize fearful and neutral faces presented at 15° and 30° to the left or right of fixation. Their findings showed a decrease in behavioral performance with eccentricity, and more importantly that fearful faces induced shorter reaction times than neutral faces. In a subsequent study, Rigoulot et al. (2012) investigated whether the implicit processing of faces in the far periphery could be modulated by their emotional expression. They presented happy, fearful and neutral faces also in the periphery (15° and 30°). Participants had to categorize the gender of the faces (female/male) and both accuracy and reaction times were recorded. They found decreased accuracy and longer reaction times for emotional faces compared to neutral faces, thus they argue that their findings demonstrate that emotional facial expressions are automatically processed even in impoverished conditions of vision. However, it remains unknown whether facial expressions can influence our perception of other facial cues (such as eye gaze) in the periphery. A recent study examining peripheral processing of gaze found that the CoDG increased for peripherally presented stimuli compared to foveally viewed ones (Palanica and Itier, 2011; Florey et al., 2015), using neutral faces only.

The aim of this experiment is to examine how the emotional expression of a face, viewed foveally or in the periphery, influences how we process information about its gaze direction. We focus here on the expression of anger since it has been shown to consistently modulate performance across a number of different tasks. We will use the CoDG to measure gaze perception in the fovea and periphery for neutral and angry faces that are viewed either on their own or within a group of other faces. We expect that angry expressions will influence performance more in the periphery than in the fovea and that as a result, the CoDG will be larger for angry faces in the periphery than in the fovea. We also expect that flanker faces will lead to contextual effects and crowding, with effects more pronounced in the periphery than in the fovea for congruent facial emotions (between target and flankers) than incongruent facial emotions.

## Materials and Methods

### Participants

Fourteen participants were included in this study (2 males), ranging from age 21 to 48 years (*M* = 28; *SD* = 7.2). All participants had normal or corrected to normal vision. The experiment was approved by the ethics board of Queen Mary University of London, and participants gave written informed consent to take part in the study. Participants received a monetary compensation. Three participants were excluded because they failed to perform above chance in the peripheral presentation experiment.

### Stimuli and Apparatus

Faces: The stimuli consisted of four synthetic grayscale faces that were generated using Daz software^1^. Two male and two female faces were used throughout the experiment, all the faces were forward facing, and either had a neutral or angry expression. In order to vary eye gaze, the original eyes of the faces were removed and replaced with grayscale eye stimuli created using Matlab that allowed us to control the horizontal and vertical gaze deviations down to the nearest pixel. For each face, the following sequence was carried out: Each forward facing stimulus uploaded into Facegen was saved as an image file and opened in Photoshop. We took note of the position of the iris and pupils and then cut out the eyes in Photoshop. We then created iris and pupil stimuli in Matlab and positioned them at the location of the original eyes in the Facegen stimulus. The inter-ocular distance was kept the same as the original faces. We wrote Matlab functions that allowed us to move the eyes following a rotational trajectory on a 3d round surface.

**FIGURE 1:**
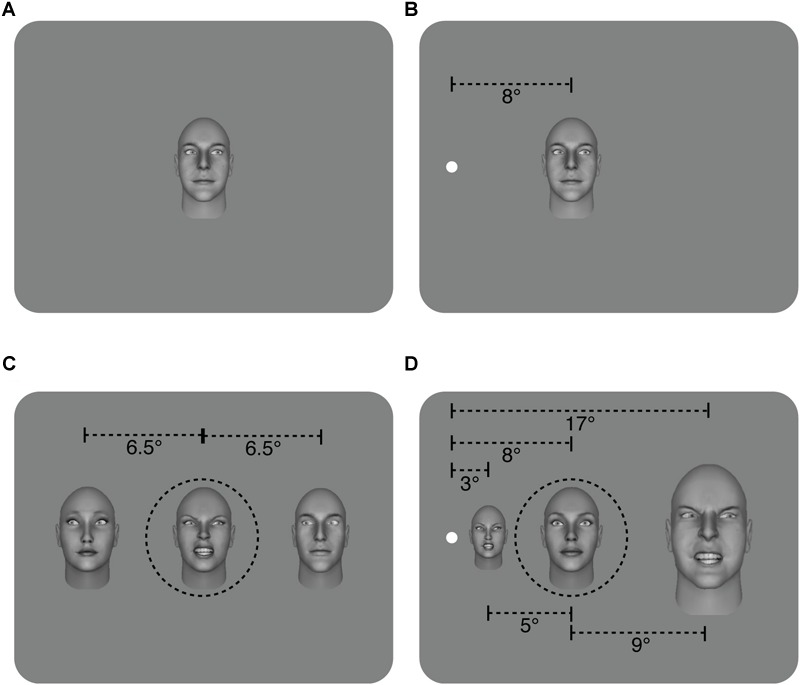
Illustration of the different conditions. **(A)** Sample of non-flanked stimuli in the foveal and, **(B)** peripheral experiments with a target face displaying a 20° rightwards gaze deviation. **(C)** Sample of the flanked stimuli in the foveal experiment. The target and two flanker faces were the same size and subtended approximately 5.5° × 8°. In this example, flanker faces have a leftwards gaze deviation of -20°, and the target face has a rightwards gaze deviation of 20°. The flankers were presented approximately 6.5° from the target (center to center spacing) **(D)** Sample of flanked stimuli in the peripheral experiment. The distance from the center of the target face to the fixation point is 8° while the inner flanker face was 5° from the target and the outer flanker face was 9° away (center to center spacing) and flanker faces have been M-scaled. Flanker faces have 20° rightwards gaze deviation while the target face has a direct (0°) gaze deviation. For simplicity, leftwards gaze deviations are assigned negative values (e.g., -20°), while rightwards gaze deviations have positive values (e.g., 20°). The target face was always in the middle of the three faces (shown here with dashed black line for illustrative purposes only – no line was present in the experiment. Also note that separations here are not shown to scale).

Configuration: In the foveal experiment (eccentricity = 0°), face stimuli were all the same size and subtended approximately 5.5° × 8°. In the peripheral experiment, The face stimuli in the peripheral experiment were M-scaled (Figure 1) using the formula from Duncan and Boynton (2003): 1/M = 0.065E + 0.054, where M is the scaling factor and E is eccentricity and subtended approximately 3.25° × 2.5° for the nearest flanker, 8° × 5.5° for the target and 17° × 11° for the further flanker. The fixation point was not presented centrally but rather 8° from the left edge of the monitor with the participant seated in front of the fixation point. We have previously found no difference between stimuli presented to the left or to the right of fixation (Florey et al., 2015), therefore we moved the fixation point to the left side of the monitor to ensure that the M-scaled stimuli could be properly displayed in the peripheral experiment. Participants were always seated in front of the fixation point and stimuli were presented in the right visual field (to the right of the fixation point). Note that in the peripheral experiment, part of the inner and outer flanker faces fell outside the traditional crowding zone defined by Bouma’s law (Pelli and Tillman, 2008). In an initial pilot, we positioned the flankers closer to the target (eccentricities of 4.2° and 14.5°, resulting in a center to center spacing with the target of 4.1° and 6.75° respectively) and in all observers (6/6), their abilty to discriminate the most extreme gaze deviations in the target face fell to chance (see Pilot section in Results).

Stimulus presentation and response collection was controlled by a DELL PC running Matlab software (MathWords Ltd.) with Psychtoolbox (Brainard, 1997). The stimuli were presented on Iiyama vision master PRO 520 monitor (1600 × 1200 pixels, 60 Hz refresh rate). At the viewing distance of 57 cm, 1 pixel subtended 1.5 arcmin. Participants were in a dimly lit room and used a chinrest during the experiment.

### Procedure

Participants took part in a gaze categorization task, they were required to judge the direction of gaze of a target face, indicating whether the eye gaze direction was averted to the left, direct (forward facing), or averted to the right using the keyboard presses “j,” “k,” “l,” respectively. The target face’s gaze deviation was randomly selected from 9 possible deviations spanning from 20° to the left to 20° to the right, in steps of 5° (-20°, -15°, -10°, -5°, 0°, +5°, +10°, +15°, +20°). Target faces were either neutral or angry and each target gaze deviation was repeated 10 times for the neutral target and angry target conditions, resulting in 180 trials.

In the flanked condition, the target face was surrounded by two flanker faces that were arranged horizontally on either side of it. Both flanker faces had the same gaze deviation that was randomly selected from 5 possible deviations (-20°, -10°, 0°, +10°, +20°) and all flanker gaze deviations were presented an equal number of times. In addition, in the congruent conditions, the target and flanker faces could be either all neutral or all angry. In the incongruent conditions, the target face could be neutral and the flankers angry or the target could be angry and the flankers neutral. Each combination of target face gaze deviation, flankers’ gaze deviation, target-face emotion and flankers’ emotion was repeated 10 times using a method of constant stimuli resulting in 1800 trials. In the non-flanked conditions, each target gaze deviation was repeated 10 times for the neutral target and angry target conditions, resulting in an additional 180 trials. The flanked and non-flanked conditions were randomly interleaved resulting in a total of 1980 trials.

In order to investigate the effect of stimulus eccentricity on gaze perception, we ran the above procedure for the two eccentricity experiments separately.

(a) In the foveal experiment, the target was presented at the central fixation (0° eccentricity) and when it was flanked, the flankers were presented approximately 6.5° from the target (center to center spacing). Flankers were the same size as the target face (5.5° × 8°).
(b) In the peripheral experiment (8° eccentricity) participants sat in front of a fixation point located on the left side of the monitor (8° from the monitor edge) and stimuli were always presented to the right of the fixation dot (to ensure they could be fully displayed on the monitor). The target face was always located 8° from the fixation dot, while the inner flanker face was 5° from the target and the outer flanker face was 9° away (center to center spacing). To ensure participants could perform the peripheral task, they completed it prior to the foveal experiment. The foveal and peripheral experiments were run separately and on separate days, but all other conditions were randomly interleaved and run in three separate blocks, with equal numbers of male and female faces used throughout.

**FIGURE 2:**
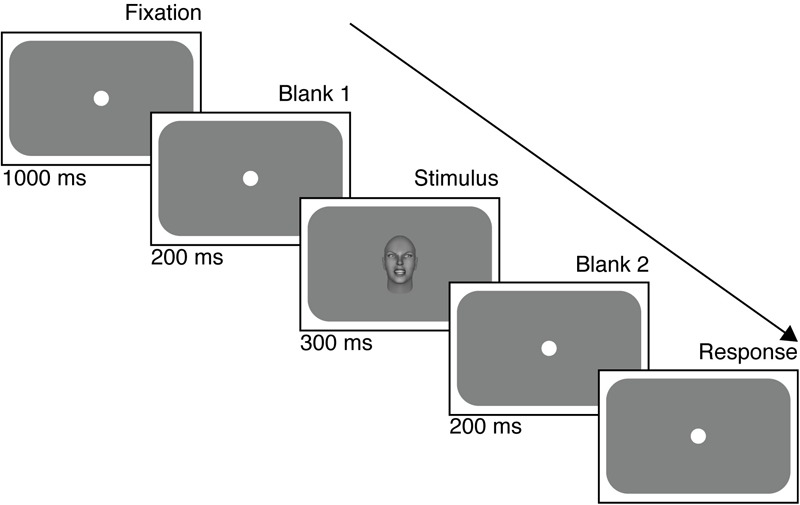
Timeline of a single trial, here showing an angry target face in the foveal experiment.

Figure 2 illustrates the timeline of a trial in the foveal experiment (a similar timeline occurred in the peripheral experiment). Each trial began with a gray screen with a central fixation point for 1000 ms, followed immediately by the stimulus for 300 ms. After the extinction of the stimulus, a gray screen was presented again for a 200 ms wait period during which time no response was recorded. The next trial was initiated only after a response was recorded following the wait period. The fixation dot (10 pixels diameter) was present throughout in the peripheral condition, apart from the response collection screen. Reaction times were not measured. In the foveal condition, the fixation dot disappeared only when the stimulus appeared in the center of the screen to avoid overlap.

### Data Analysis

**FIGURE 3:**
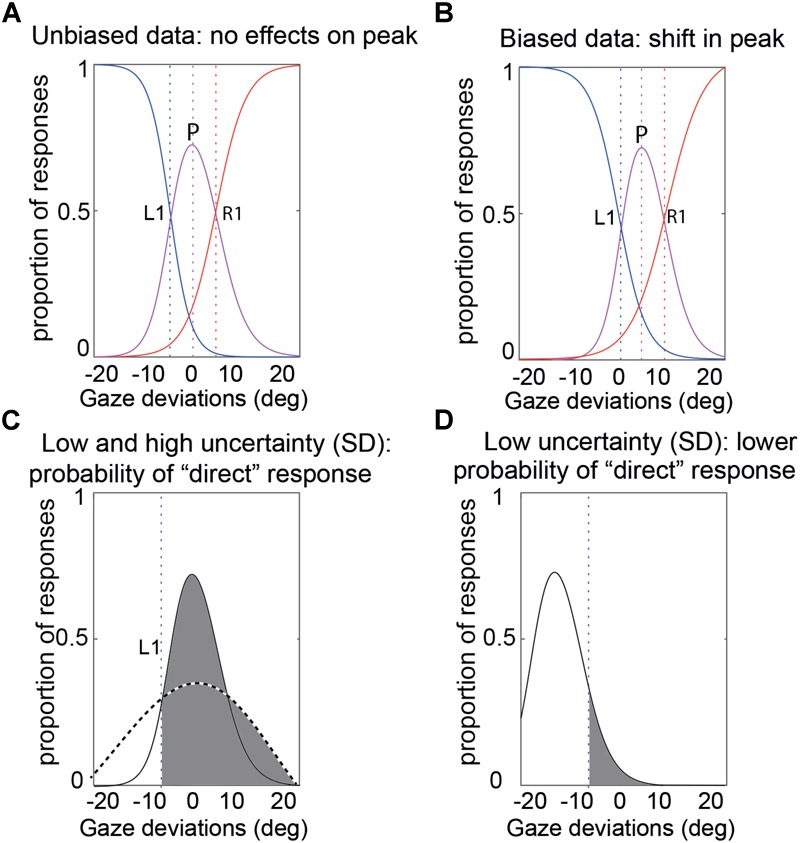
Illustration of the CoDG model parameters. **(A)** Sample unbiased data. The bias (peak) is at 0. The CoDG is the distance between R1 and L1 **(B)** Sample biased data. The bias (peak) is roughly 2° to the right of 0°. **(C)** Standard deviation. This represents the noise of the sensory representation associated with a gaze deviation (here shown for a gaze deviation of 0°). The dashed curve illustrates a high SD and the solid curve a low SD, and the vertical dashed line represents the left category boundary. In the case of the high SD, a stimulus at 0° will most likely elicit a “direct” response (area shaded in gray) whereas in the case of the low SD, there is a greater chance that it may elicit a “left” response. **(D)** Example of a low SD, a -15° stimulus has a narrow sensory representation and the probability of a direct response is low (area in gray).

In order to quantify participants’ gaze perception across the different conditions in the foveal and peripheral experiments, the data from separate conditions were compiled into the proportion of “left,” “direct,” and “right” responses and we fitted our psychophysical model to each participant’s data (Mareschal et al., 2013b). As mentioned earlier, the model has three parameters (see Figure 3) to account for an observer’s performance: (1) the peak of the CoDG or the bias of perceived direct gaze. This is the gaze deviation that participants judge most as being direct and is 0 if there is no bias, and significantly different from zero if there is a bias. For example, in the case of a *rightwards* bias, participants perceive a physically direct gaze as being rightwards and therefore a *leftwards* gaze as being direct. Hence the sign of the peak parameter reflects the direction of the bias; (2) the boundary width (akin to the CoDG). Gaze judgments have been shown to arise from a three-channel process (a channel processing leftwards gaze deviations, one processing direct gaze deviations and one processing rightwards gaze deviations- e.g., Calder et al., 2007, 2008). The boundary width therefore represents the width between the categorical boundaries between the averted gaze deviations and direct gaze and reflects the range of gaze deviations that participants judge as being direct (e.g., Figure 5, 6); (3) the standard deviation of participants’ sensory representation of gaze. This represents the noise of the sensory representation associated with the gaze deviation. If the SD is high, this means that the same gaze deviation may elicit different responses from the observer (e.g., sometimes “left”; sometimes “direct”), whereas if the SD is low, the gaze deviation will elicit the same response from the observer. This estimate therefore reflects the (sensory) noise associated with the gaze perception process.

In order to examine the effects of eccentricity, flankers’ eye gaze, and emotional content of both the target face and the flankers on the perception of the eye gaze of the target face, we performed a 5 (surround gaze deviation) × 2 (target emotional expression) × 2 (flankers’ emotional expression) × 2 (eccentricity) repeated measures, four-way analysis of variance (ANOVA) on each of the three CoDG model parameters. We report results as significant at α = 0.05.

## Results

### Pilot

**FIGURE 4:**
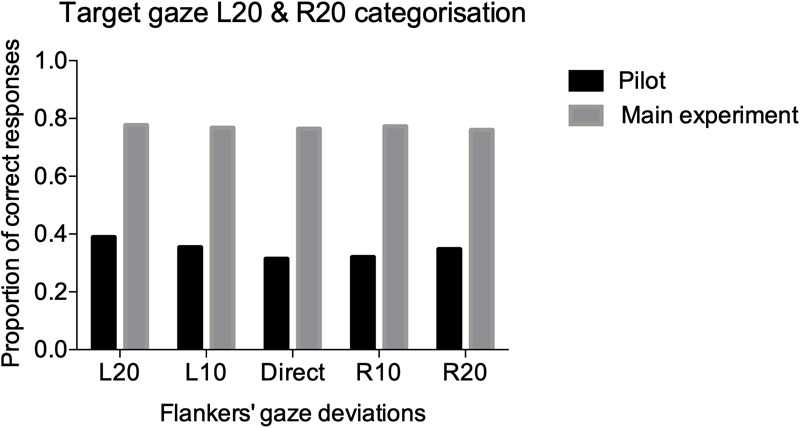
Performance accuracy for the pilot (black), and main (gray), experiments. We summed the correct responses (e.g., left response for L20 target and right response for R20) for the two extreme gaze deviations L20 and R20 across all participants for the five different flanked conditions.

Six participants performed the task for the periphery pilot. The CoDG model was fit to the averaged data across the six participants because it failed to fit the individual data in 4/6 participants (e.g., logistic functions failed to fit the data because responses across the different deviations were around 50%, even for the extreme deviations where the responses should have been close to 100 or 0%). Therefore, we calculated the proportion of correct responses at the extreme deviations in the averaged fits (e.g., L20 stimulus and “leftwards” response or R20 stimulus and “rightwards” response) and summed these across all flanker conditions. The extreme gaze deviations are the least ambiguous amongst the different gaze deviations (i.e., L20 is more clearly leftward gaze than L5), therefore, it is expected that gaze judgments should have the highest correct responses in those trials. However, data in Figure 4 show that observers performed at chance for all flanker conditions (we include for comparison, percent correct performance on the main task from the next section).

### Experiment

**FIGURE 5:**
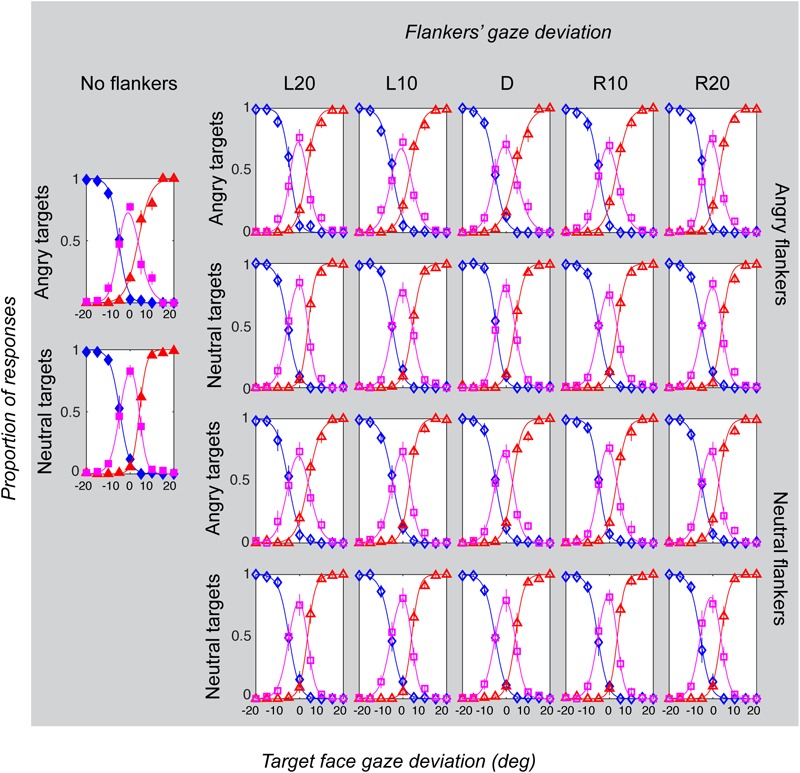
Gaze categorization results for the foveal experiment across all conditions, averaged across all participants. The proportion of “leftwards” (blue diamonds), “direct” (pink squares) and “rightwards” (red triangles) responses are plotted as a function of the target gaze deviation. The top two rows plot the two angry flankers conditions, and the bottom rows plot the two neutral flankers conditions. Each column represents one of the six flanker conditions (No flankers corresponds to the unflanked condition, followed by flankers with the five different deviations: L20 = -20°, L10 = -10°, direct = 0°, R10 = 10°, R20 = 20° (negative values = leftward). Error bars represent 1 ± SEM.

**FIGURE 6:**
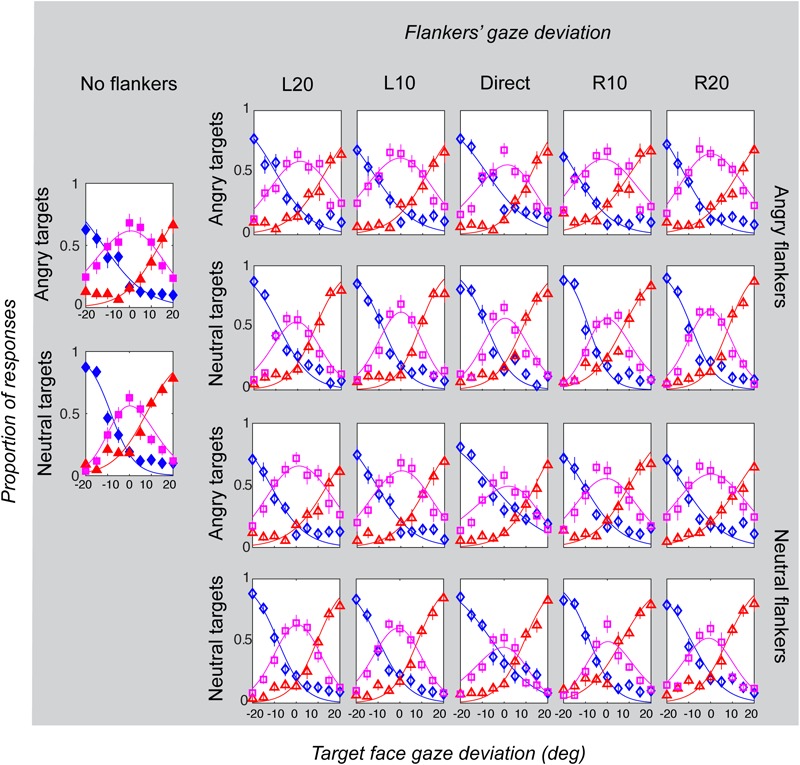
Gaze categorization results for the peripheral experiment across all conditions, averaged across all participants. The proportion of “leftwards” (blue diamonds), “direct” (pink squares) and “rightwards” (red triangles) responses are plotted as a function of the target gaze deviation. The top two rows plot the two angry flankers conditions, and the bottom rows plot the two neutral flankers conditions. Each column represents one of the six flanker conditions (No flankers corresponds to the unflanked condition, followed by flankers with the five different deviations: L20 = -20°, L10 = -10°, direct = 0°, R10 = 10°, R20 = 20° (negative values = leftward). Error bars represent 1 ± SEM.

**FIGURE 7:**
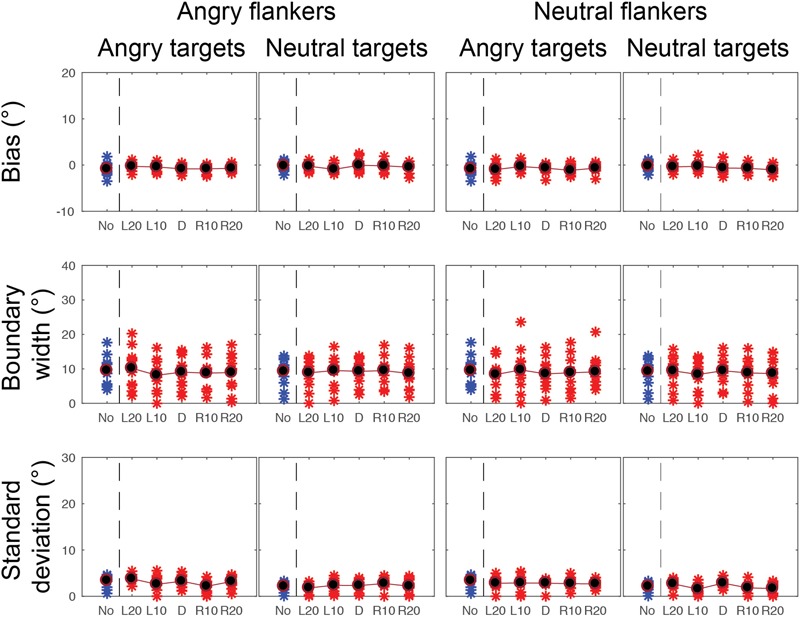
CoDG model parameters across all conditions in the foveal experiment. (top) Estimates of peak or bias, (middle) boundary width, (bottom) standard deviation, or uncertainty. Parameter values are plotted for each participant (red crosses) across the five different flankers’ eye gaze deviations with blue crosses for non-flanked condition (No). Averaged data are in black.

**FIGURE 8:**
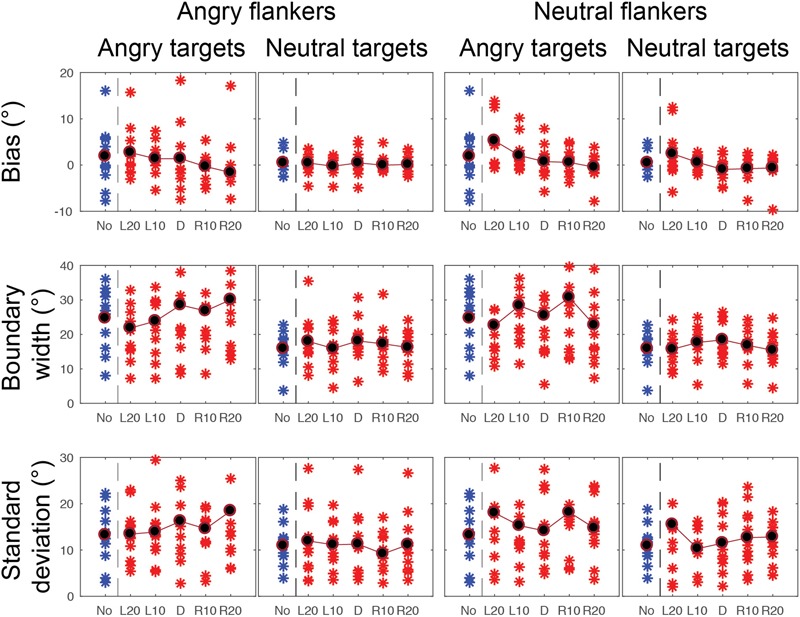
CoDG model parameters across all conditions in the peripheral condition. (top) Estimates of peak or bias, (middle) boundary width, (bottom) standard deviation, or uncertainty. Parameter values are plotted for each participant (red crosses) across the five different flankers’ eye gaze deviations with blue crosses for non-flanked condition (No). Averaged data are in black.

Gaze categorization results are plotted in Figure 5 for the foveal experiment and Figure 6 for the peripheral experiment. Estimates of peak (bias), boundary width and standard deviation from the CoDG model are plotted in Figure 7 for the foveal experiment and Figure 8 for the peripheral experiment. Below we present the results for the ANOVA on each of the CoDG model parameters separately.

#### Bias (Peak)

We sought to determine the peak gaze deviation participants judged most as being direct (their bias), and whether this value significantly differed from 0 as a function of the experimental conditions. For example, if the flankers’ eye gaze direction influences perception of the target’s gaze, this would be reflected by systematic shifts in the bias or peak. Specifically, if information about the target and flankers is being averaged, we would expect that the peak gaze deviation that participants’ judge as being direct would be shifted *away* from the flankers gaze deviation (e.g., if the flankers are *leftwards*, L20) and there is averaging with the target, a physically direct target gaze would be judged as leftwards, and therefore the peak of direct responses would occur for a *rightwards* deviated target).

We found a significant main effect of eccentricity on the bias parameter *F*(1, 10) = 5.99, *p* = 0.034, $\eta_{p}^{2}$ = 3.41, revealing a significant difference between the biases in the foveal condition (*M* = -0.58, *SD* = 0.21) and the peripheral condition (*M* = 0.66, *SD* = 0.47). Mauchly’s test indicated that the assumption of sphericity had been violated for flankers’ gaze deviation conditions, X^2^(9) = 19.66, *p* = 0.027, therefore Greenhouse-Geisser corrections are reported. We found a significant main effect of flankers’ gaze deviation on the peak of the CoDG, *F*(2.4, 24.02) = 7.60, *p* = 0.002. There was no significant effect of the target emotional expression *F*(1, 10) = 0.37, *p* = 0.559, $\eta_{p}^{2}$ = 0.96 or the flankers’ emotional expression *F*(1, 10) = 0.162, *p* = 0.696, $\eta_{p}^{2}$ = 0.43 on the peak of the CoDG.

There was a significant interaction between eccentricity and flankers’ eye gaze deviations *F*(2.5, 10) = 6.34, *p* = 0.004. To explore this interaction, we conducted a series of paired-sample *t*-tests between each flanker’s eye gaze deviation in the foveal experiment with the corresponding gaze deviation in the peripheral experiment. After Bonferroni correction, we found a significant difference in the peak *t*(10) = 4.508, *p* = 0.005 between the flanker’s eye gaze deviation L20 in the periphery (*M* = 2.71, *SD* = 2.51) and the fovea (*M* = -0.47, *SD* = 0.74). We also found a significant difference in the peak *t*(10) = 3.363, *p* = 0.036 between the flanker’s eye gaze deviation L10 between the peripheral (*M* = 0.94, *SD* = 0.90) and foveal condition (*M* = -0.48, *SD* = 0.73). No other comparisons were significant (*p* > 0.05). As expected with averaging, the peak was shifted away from the flankers’ gaze deviation although this effect was only significant for L20 and L10 and showed a trend in the predicted direction for R10 (*M* = -0.13, *SD* = 1.93) and R20 (*M* = -0.65, *SD* = 1.91).

In order to investigate whether there was a significant effect of the presence of flankers on the peak of the CoDG between the unflanked condition and the flanked conditions with the five different flankers gaze deviations, we ran a separate one-way ANOVA with six levels (5 flanker gaze deviations and the no surround condition). We found a significant difference between the six conditions *F*(2.63, 26.27) = 5.599, *p* = 0.006, however, following Bonferroni correction, none of the paired *t*-tests were significant (*p* > 0.05).

#### Boundary Width

We sought to determine whether the range of gaze deviations that participants judged as direct differed across the conditions. We found a significant main effect of eccentricity on the boundary width *F*(1, 10) = 38.307, *p* = 0.000, $\eta_{p}^{2}$ =6.22, revealing a significantly narrower boundary width in the foveal experiment (*M* = 9.012, *SD* = 1.321) than in the peripheral experiment (*M* = 21.515, *SD* = 2.520). There was also a significant main effect of target emotion on the boundary width, *F*(1, 10) = 9.185, *p* = 0.013, $\eta_{p}^{2}$ =2.39, revealing a significantly wider boundary width for an angry target (*M* = 17.528, *SD* = 2.388) than for a neutral target (*M* = 13.001, *SD* = 1.213). There was also a significant main effect of flankers’ gaze deviation on the boundary width, *F*(4, 40) = 3.371, *p* = 0.018, $\eta_{p}^{2}$ =0.252. However, further *t*-tests revealed that the only significant comparisons were in the peripheral experiment between flankers’ gaze deviation L20 (*M* = 19.52, *SD* = 7.17) and flankers’ direct gaze deviation (0) (*M* = 22.63, *SD* = 9.61) *t*(10) = -2.26, *p* = 0.047, $\eta_{p}^{2}$ = 0.37 and between L20 (*M* = 19.5, *SD* = 7.17) and R10 (*M* = 22.21, *SD* = 9.67) *t*(10) = -2.66, *p* = 0.024, $\eta_{p}^{2}$ = 0.31. Lastly there was no significant effect of the flankers’ emotion on the boundary width *F*(1, 10) = 0.161, *p* = 0.696, $\eta_{p}^{2}$ = 0.12.

There was a significant interaction between eccentricity and target emotion on the boundary width *F*(1, 10) = 11.461, *p* = 0.007. To explore this interaction further, we ran paired *t*-tests between the different target emotion conditions in the fovea and the periphery. After Bonferroni correction, we found that the boundary width was significantly wider *t*(10) = 5.17, *p* = 0.00, $\eta_{p}^{2}$ = 1.81 for an angry target presented in periphery (*M* = 26.09, *SD* = 12.63) than a neutral target presented in fovea (*M* = 9.06, *SD* = 4.21), and significantly wider *t*(10) = 5.98, *p* = 0.00, $\eta_{p}^{2}$ = 1.66 for a neutral face presented in periphery (*M* = 16.94, *SD* = 4.86) than an angry face presented in fovea (*M* = 8.97, *SD* = 4.72). Furthermore, we also found that in the periphery, the width of the CoDG was significantly wider *t*(10) = 3.26, *p* = 0.01, $\eta_{p}^{2}$ = 0.96 for angry faces (*M* = 26.09, *SD* = 12.63) than neutral faces (*M* = 16.94, *SD* = 4.86). There was no significant difference between angry and neutral faces in fovea *t*(10) = -01.17, *p* = 0.87.

There was a significant interaction between eccentricity and flankers’ eye gaze on the width of the CoDG *F*(4, 40) = 3.252, *p* = 0.021. Paired *t*-tests with Bonferroni corrections revealed that there was a significant difference in the CoDG width between the flankers’ eye gaze deviation L20 *t*(10) = 5.90, *p* = 0.00, $\eta_{p}^{2}$ = 1.71, in the periphery (*M* = 19.52, *SD* = 7.17) and the fovea (*M* = 9.21, *SD* = 4.60). We also found a significant difference for flankers’ eye gaze deviation L10 *t*(10) = 6.49, *p* = 0.00, $\eta_{p}^{2}$ = 1.93, in the periphery (*M* = 21.44, *SD* = 7.82) compared to the fovea (*M* = 8.94, *SD* = 4.73), a significant difference between the flankers’ direct eye gaze *t*(10) = 5.51, *p* = 0.00, $\eta_{p}^{2}$ = 1.84, in the periphery (*M* = 22.63, *SD* = 9.60) and the fovea (*M* = 9.10, *SD* = 4.06), a significant difference between the flankers’ eye gaze R10 *t*(10) = 6.01, *p* = 0.00, $\eta_{p}^{2}$ = 1.85 in the periphery (*M* = 22.91, *SD* = 9.67) and the fovea (*M* = 8.90, *SD* = 4.58). Finally, a significant difference between the flankers’ eye gaze R20 *t*(10) = 5.68, *p* = 0.00, $\eta_{p}^{2}$ = 1.78, in the periphery (*M* = 21.10, *SD* = 8.56) and the fovea (*M* = 8.84, *SD* = 4.58).

In order to investigate whether there was a significant effect of the presence of flankers on the width of the CoDG between the unflanked condition and the flanked conditions with the five different flankers gaze deviations, we ran a separate one-way ANOVA with six levels (5 flaker gaze deviations and the no surround condition). However, there was no significant difference between the different conditions *F*(5, 50) = 1.78, *p* = 0.134.

#### Participants’ Standard Deviation (SD)

We found a significant main effect of eccentricity on the SD *F*(1, 10) = 31.516, *p* = 0.00, $\eta_{p}^{2}$ = 7.96, indicating that the SD was lower in the foveal experiment (*M* = 2.542, *SD* = 0.141) than in the peripheral experiment (*M* = 13.714, *SD* = 1.979). We also found a significant main effect of target emotion on the SD *F*(1, 10) = 31.346, *p* = 0.000, $\eta_{p}^{2}$ =2.29, revealing that the SD was significantly greater in the angry target condition (*M* = 9.286, *SD* = 1.119), than the neutral target condition (*M* = 6.969, *SD* = 0.888). There was no significant effect of the flankers’ emotion *F*(1, 10) = 1.169, *p* = 0.305 or flankers’ gaze deviation *F*(4, 40) = 1.030, *p* = 0.404 on the SD.

There was a significant interaction between eccentricity and target emotion on the SD *F*(1, 10) = 14.761, *p* = 0.003. To further explore this interaction, we ran paired *t*-tests between the different target faces’ emotions and the different eccentricities. After Bonferroni correction, we found that the SD was different in the periphery according to the emotional content of the target face *t*(10) = 5.003, *p* = 0.03, $\eta_{p}^{2}$ = 0.60, revealing that the SD was greater for angry targets in periphery (*M* = 15.69, *SD* = 7.42) than neutral targets in the periphery (*M* = 11.74, *SD* = 5.88). The SD was also significantly higher *t*(10) = 5.67, *p* = 0.00, $\eta_{p}^{2}$ = 2.43, for angry targets in periphery (*M* = 15.69, *SD* = 7.42) compared to angry targets in the fovea (*M* = 2.89, *SD* = 0.69). The SD also significantly increased *t*(10) = 5.96, *p* = 0.00, $\eta_{p}^{2}$ = 2.56, for angry targets in periphery (*M* = 15.69, *SD* = 7.42) compared to neutral targets in fovea (*M* = 2.20, *SD* = 0.63). The SD also significantly increased *t*(10) = 5.00, *p* = 0.00, $\eta_{p}^{2}$ = 2.11 for neutral targets in periphery (*M* = 11.74, *SD* = 5.88) compared to angry targets in fovea (*M* = 2.89, *SD* = 0.69). And lastly, the SD significantly increased *t*(10) = 5.34, *p* = 0.00, $\eta_{p}^{2}$ = 2.28 for neutral targets in the periphery (*M* = 11.74, *SD* = 5.88) compared to neutral targets in fovea (*M* = 2.20, *SD* = 0.63). We found no significant difference in SD between angry and neutral faces in fovea *t*(10) = 2.43, *p* = 0.21.

To investigate whether there was a difference on the SD between the unflanked condition and the flanked conditions with the five different flankers gaze deviations, we ran a separate one-way ANOVA with six levels (5 flanker gaze deviations and the no surround condition). However, there was no significant difference between the different conditions *F*(5, 50) = 1.208, *p* = 0.319.

To summarize our main results (see Table 1 for summary of significant main results), we found an effect of eccentricity on perception of gaze, across all three model parameters. In the peripheral experiment, participants categorized gaze as direct over a wider range of gaze deviations than in the foveal experiment, across all conditions. When we examined for target emotion, we found that angry faces led to a significant increase in the boundary width and SD for angry targets in the periphery only. There was no effect of the flanker’s emotional expression on target gaze categorization. We report weak evidence for the flankers’ gaze deviation affecting perception of the target’s gaze for leftwards gazing flankers only (peak shifts with L20 and L10, with a trend in the expected direction for R10 and R20), although there was no significant difference between the flanked and unflanked conditions.

**Table 1:** Summary of significant main effects and interactions between the different experimental conditions and the model parameters.

Effect	Bias (peak)	Boundary Width	Standard deviation
Eccentricity	*Significant: F*(1, 10) = 5.99, *p* = 0.034, $\eta_{p}^{2}$ = 3.41	*Significant: F*(1, 10) = 38.307, *p* = 0.000, $\eta_{p}^{2}$ =6.22	*Significant: F*(1, 10) = 31.516, p = 0.00, $\eta_{p}^{2}$ = 7.96
Target emotion		*Significant: F*(1, 10) = 9.185, *p* = 0.013, $\eta_{p}^{2}$ =2.39	*Significant: F*(1, 10) = 31.346, *p* = 0.000, $\eta_{p}^{2}$ =2.29
Flankers’ eye gaze	*Significant: F*(2.4, 24.02) = 7.6, *p* = 0.002	*Significant: F*(4, 40) = 3.371, *p* = 0.018, $\eta_{p}^{2}$ =0.252.	
Eccentricity ^∗^ Target emotion		*Significant: F*(1, 10) = 11.461, *p* = 0.007	*Significant: F*(1, 10) = 14.761, *p* = 0.003.
Eccentricity ^∗^ Flankers’ eye gaze	*Significant: F*(2.5, 10) = 6.339, *p* = 0.004	*Significant: F*(4, 40) = 3.252, *p* = 0.021	

*Please refer to the results discussion for further details on t-tests when applicable.*

## Discussion

We examined whether the emotional expression of a face influences how we process information about its direction of gaze when viewed directly (foveal experiment) or not (peripheral experiment). We found that gaze perception depends on both the eccentricity and the emotional expression of the face being evaluated. Specifically, we found that observers judge a target face’s gaze as being direct over a broader range of gaze deviations when the faces are presented in the periphery than in the fovea, consistent with previous findings (Florey et al., 2015). We report that angry faces are judged as looking direct over a wider range of gaze deviations than neutral faces when they are in the periphery only. Although we found some evidence that the presence of flankers affects performance by shifting the peak of the CoDG, there was no effect of flankers’ emotion on the task.

### Facial Emotional Effect

We found that angry target faces lead to a wider CoDG than neutral target faces, for faces presented in the periphery only. Although a wider CoDG with angry faces in the periphery is consistent with previous suggestions that threat stimuli elicit attention, our foveal result is at odds with earlier reports that faces displaying angry expressions are more likely to be perceived as looking direct when viewed centrally (Lobmaier et al., 2008; Ewbank et al., 2009). However, more recent results using highly anxious populations have found an increased tendency to orient a person’s gaze direction for faces showing fearful or angry expression for anxious participants compared to non-anxious participants (Mathews et al., 2003; Holmes et al., 2006). The role of participant anxiety has been more recently linked to the CoDG where Jun et al. (2013) found that the width of the CoDG increased for anxious males only. Therefore, our results are in line with Jun et al. (2013), since the majority of our participants were female. However, given that we do find an effect of target emotion for stimuli in the periphery, this would suggest that the processing of emotional faces not viewed directly does not depend on gender or (potential) participant anxiety, perhaps reflecting a generic mechanism to respond to threat.

Why should the CoDG increase with facial emotion in the periphery? Eye gaze generally indicates that we are the object of another person’s attention (Cary, 1978). If that person is displaying an angry expression this would be indicative of a threatening situation, so it might be beneficial to assume they are looking at us. Therefore, when the gaze is close to direct (for example 5° averted to the left or to the right), falsely perceiving that an angry face is looking at oneself is less costly and less dangerous than falsely missing it and ignoring an alarming threat. This is consistent with previous findings that angry emotional expressions and direct gaze are enhanced by approach-avoid self-preservation motivations (Adams and Kleck, 2005; Rigoulot et al., 2012). This is also in line with previous studies demonstrating that the perceived intensity of an angry face is increased when it displays direct gaze (Adams and Kleck, 2005). Taken together, this suggests that participants’ sense that gaze is directed toward them with angry faces should increase because of the potential threat this stimulus represents. Emotional scenes (Calvo and Lang, 2005) and emotional faces (Rigoulot et al., 2012) are processed more quickly in peripheral vision suggesting an attention-grabbing mechanism, which is consistent with studies showing that it takes longer to disengage spatial visual attention from threatening stimuli compared to neutral stimuli (Stein et al., 2009). Rigoulot et al. (2012) propose that this attentional capture is due to the necessity to react to relevant stimulations (both negative and positive) in peripheral vision, even if attention is not consciously directed toward them. For example, Calvo and Lang (2005) investigated whether emotional visual scenes are more likely to attract a person’s eye movements than neutral scenes. Pairs of emotional (either pleasant scenes such as people enjoying themselves or non-pleasant scenes such as people suffering harm) and neutral scenes (people performing a variety of daily activities) were presented parafoveally (2.1° or 2.5° from a fixation point) for 150–3000 ms, followed by an immediate recognition test (500 ms delay). They found that when the emotional and neutral scenes were presented simultaneously in parafoveal vision, the eyes moved to and fixated the emotional scene rather than the neutral scene, revealing initial orienting toward the emotional stimuli. Furthermore, the authors suggest that the meaning or content of the emotional scenes drew overt attention that is responsible for this early orienting effect.

However, it is also worth noting that angry faces are physically different from neutral faces that may increase their ambiguity. For example, the opening of the eyes in angry faces is smaller as a result of the furrowing of the brows. It is possible that those physical facial differences may have increased the ambiguity of the angry faces, leading to an increase in direct gaze judgments. Previous research has shown that we tend to judge gaze as being directed at us when it is ambiguous (Mareschal et al., 2013a, 2014), We find here that the SD is higher for angry faces in the periphery, consistent with the idea that participants were more uncertain in this condition, it is possible that the target’s gaze was more ambiguous in this condition and that this leads to an increase in direct responses.

### Eccentricity Effect

We found that faces presented in the periphery led to an increased number of direct responses over a wider range of eye gaze deviations (a wider CoDG), consistent with previous results (Florey et al., 2015), and demonstrating that participants held fixation. This suggests that people assume they are being looked at when they are not directly viewing the faces, consistent with our findings here of increased uncertainty when making gaze judgments about faces in the periphery. One explanation for this increase in direct responses for faces in the periphery is that it is beneficial to assume that we are being looked at, notably in the case of threatening stimuli (e.g., Mareschal et al., 2013a, 2014). This decrease in accuracy of behavioral judgments (wider CoDG) with eccentricity is consistent with earlier results (Thorpe et al., 2001; Rigoulot et al., 2011, 2012). For example, Rigoulot et al. (2012) investigated implicit emotional processing in peripheral vision, and found a gradual decrease of behavioral performance with eccentricity, revealed by the lower rate of correct categorization and longer reactions times. However, it is important to note that their results also showed that emotional facial expressions could be automatically detected in peripheral vision (which is consistent with our target face emotional effect results discussed above). Similarly, Thorpe et al. (2001) investigated performance in peripheral vision by requiring participants to categorize pictures presented at different eccentricities. In their study, the authors found a linear decrease of performance with increasing eccentricity, supporting the suggestion that we make less accurate judgments about objects or faces when they are presented in the periphery.

### Averaging

We expected to find significant differences in participants’ bias across the different surround gaze conditions for stimuli presented in the periphery. We found that the peak of the direct responses for the target gaze categorization (their bias) shifted toward the gaze deviation of the surrounding flankers, reflecting averaging between the target gaze deviation and the flanker’s gaze deviation, suggesting that participants were not ignoring the flankers. However, we also surprisingly found no difference in performance between the flanked and unflanked conditions. The lack of a difference between the unflanked and the flanked conditions suggests no crowding for gaze judgments. However, it is important to note that we had to move the inner face flanker further toward fixation, so that it was only partially within the crowding zone to increase performance from chance. Therefore gaze direction cannot be accurately reported when flanking faces are too close to a target face, presumably due to crowding effects. This is consistent with a recent study by Kalpadakis-Smith et al. (2018) who report crowding for judgments about the spacing of the two eyes within the face. Although participants were unable to perform our gaze task when the flankers were partially within the crowding zones, this may simply reflect the fact that gaze direction judgments require finer resolution than those made by the participants in Kalpadakis-Smith et al. (2018).

Despite no difference between the flanked and unflanked conditions on gaze judgments we find significant differences in the peaks with the different flankers’ gaze deviations conditions. This could arise for a couple of reasons. First, this might reflect occasional flanker substitution (e.g., where participants erroneously report the flanker gaze deviation rather than the target’s (Estes et al., 1976; Chastain, 1982; Põder and Wagemans, 2007; Freeman et al., 2012). Although this may have occurred some of the time, we can rule out that participants consistently reported the flanker gaze deviation, since those target gaze deviations that were in opposite direction to the flankers’ deviations were rarely misclassified to the flankers’ gaze (e.g., a target face with a leftwards gaze of 20° surrounded by rightwards flankers was misclassified as rightwards 10% of the time). Alternatively, since the fixation point was presented on the left side of the monitor, and the faces were always presented to the right of the fixation point, when the flankers’ gaze was leftwards (L20 and L10), this could have been perceived by the participants as being directed toward them, leading to different CoDG peaks. This is in line with our findings that participants judged the target face as looking more direct only for flankers’ gaze deviation L20 and L10. This could also be influenced by the head orientation of the stimuli. In this experiment, all the faces were forward-facing. It has been suggested that observers first perform a symmetry judgment on faces in the periphery (forward-facing faces are symmetrical while turned faces are non-symmetrical), and that forward-facing faces are categorized as “direct” over a wider range of eye gaze deviations in the periphery in forward facing heads (Palanica and Itier, 2011; Florey et al., 2015). Second, since the participants were looking at a fixation point, and the leftward looking flankers could also be perceived as looking at the fixation point, this might resemble some form of joint attention leading to a greater number of direct responses. For example, Edwards et al. (2015) suggest that people rapidly orient their attention toward an individual with whom they have established joint attention, although this only really occurs for joint attention of a real object (not fixation points).

Finally, we found that the emotional expression of the target face significantly influenced eye gaze perception, but found no effect of flankers’ emotional expression on the categorization of the target gaze deviation. We had expected that the flankers’ emotional content could influence performance on the judgment of target gaze in two different ways. On the one hand, when the flankers and target face had the same emotional expression, we might have expected more of an influence of the flankers gaze on the perception of target gaze since crowding is most pronounced when the flankers resemble the targets (Kooi et al., 1994). On the other hand, we might expect less influence of the flankers gaze on the target’s gaze since the targets might have popped out (e.g., “gaze in the crowd effect”). We failed to find any difference, suggesting that the flankers did not pop out (this aligns with informal reports from our observers that they were unaware of the flankers’ emotions), possibly because of the attentional demands of the task at hand.

## Ethics Statement

This study was carried out in accordance with the recommendations of Queen Mary University of London (QMUL) Ethics committee. All subjects gave written informed consent in accordance with the Declaration of Helsinki. The protocol was approved by Queen Mary University of London (QMUL) Ethics committee.

## Author Contributions

DA and IM: experiment design, stimuli preparation, programming of experiment, running experiment, data analysis, and writing the manuscript. NE: experiment design and manuscript editing.

## Conflict of Interest Statement

The authors declare that the research was conducted in the absence of any commercial or financial relationships that could be construed as a potential conflict of interest.

## Footnotes

1 www.daz3d.com/
